# Risk-associated factors associated with the bovine viral diarrhea virus in dromedary camels, sheep, and goats in abattoir surveillance and semi-closed herd system

**DOI:** 10.14202/vetworld.2022.1924-1931

**Published:** 2022-08-16

**Authors:** Abdullah I. A. Al-Mubarak, Jamal Hussen, Mahmoud Kandeel, Anwar A. G. Al-Kubati, Baraa Falemban, Abdullah Skeikh, Maged Gomaa Hemida

**Affiliations:** 1 Department of Microbiology, College of Veterinary Medicine, King Faisal University, Al-Hofuf, Saudi Arabia; 2 Department of Biomedical Sciences, College of Veterinary Medicine, King Faisal University, Al-Hofuf, Saudi Arabia; 3 Department of Pharmacology, Faculty of Veterinary Medicine, Kafrelsheikh University, Kafrelsheikh, Egypt; 4 Department of Veterinary Medicine, Faculty of Agriculture and Veterinary Medicine, Thamar University, Dhamar, Yemen; 5 Camel Research Center, King Faisal University, P. O. Box 400, Al Hufuf, 31982, Al-Ahsa, Saudi Arabia; 6 Department of Veterinary Biomedical Sciences, College of Veterinary Medicine, Long Island University, Brookville, NY, 11548, USA; 7 Department of Virology, Faculty of Veterinary Medicine, Kafrelsheikh University, Egypt

**Keywords:** antibody, antigen, bovine viral diarrhea virus, dromedary camel, enzyme-linked immunosorbent assay, goats, risk factors, serosurveillance, sheep

## Abstract

**Background and Aim:**

Bovine viral diarrhea virus (BVDV) is one of the most important viral pathogens causing high economic losses in cattle of all ages. Despite the active vaccination campaigns against BVDV, many outbreaks are still detected in various populations of cattle worldwide. Other species of animals such as dromedary camels, sheep, and goats may harbor BVDV infection and cause variable clinical syndromes. Thus, they may act as a source of infection to the cattle population around them. However, little is still known about the roles of these animals in the viral transmission and sustainability of BVDV in the environment. This study aimed to explore if the dromedary camels, sheep, and goats may seroconvert against BVDV and to study some associated risk factors for BVDV in these species of animals.

**Materials and Methods:**

We tested 1012 serum samples from dromedary camels, 84 from goats, and 21 from sheep for BVDV antibodies using commercial enzyme-linked immunosorbent assay (ELISA) kits. Meanwhile, we selected 211 serum samples from dromedary camels to be tested for the BVDV antigen using the commercial ELISA kits.

**Results:**

Our results show that 49/1117 serum samples were positive for the BVDV antibodies in dromedary camels (46/1012), goats (3/84), and none of the tested sheep samples were positive. However, none of the collected serum samples tested positive for the BVDV antigen.

**Conclusion:**

Seroconversion of some dromedary camels, sheep, and goats to the BVDV with no history of vaccination against BVDV strongly suggests the potential roles of these species of animals in the virus transmission cycle. The main limitations of the current study are (1) the lack of samples from other species of animals that lived close by these animals, particularly cattle. (2) lack of follow-up samples from the same animal over a long period. We believe the long-term longitudinal study of BVDV in various species of animals, particularly dromedary camels, goats, and sheep, is one of our future research directions. This will provide more information about the dynamics of BVDV antibodies in these species of animals.

## Introduction

Although the main host for the bovine viral diarrhea virus (BVDV) is cattle, the virus can also infect other animal species such as sheep, goats, and dromedary camels [1]. BVDV infection usually results in a wide range of clinical syndromes in the affected animal spies, such as mucosal diseases, immunosuppression, reduction in milk production, decrease in feed conversion rates, and reproductive failure [2]. The main transmission routes of BVDV are the fecal-oral route and the inhalation of the contaminated materials from infected animals BVDV [3]. There are two types of BVDV infections in animals (transient or persistent) [4]. Shedding of the virus was reported in most of the body’s secretions and excretions [3]. Thus, detection of the virus particles, viral antigens, and nucleic acids can be detected in those body fluids of the affected animals. BVDV belongs to the family *Flaviviridae* and genus *Pestivirus*. There are several methods for virus classification. The most common way is mainly based on the possibility of growth, multiplication, and production of cytopathic effects on cell cultures (serotypes 1 and 2). However, the most accurate method for virus classification is genotyping based on the sequence of several regions of the viral genomes, particularly the 5’UTR, N^pro^, and E2 regions [5]. Several approaches were adopted for the laboratory viral diagnosis, including methods for the detection of viral antigen, viral antibodies, as well as viral nucleic acids [6]. Great attention was paid to studying various aspects of BVDV infection in cattle; however, little is still known about the viral infections in other species of animals, particularly the family *Camelidae* (dromedary camels and the new world camels Llamas and Alpacas) [2, 7, 8]. Some recent studies showed the possibility of a spillover of BVDV from wildlife animals to domestic animals living in their close proximity. Some recent studies reported the seroprevalence of some white-tailed deer to BVDV in the USA that lived in close contact with other livestock species of animals [9]. These findings strongly suggest that wildlife animals could act as a reservoir for the BVDV.

In the Arabian Peninsula, there is a trend of raising several animal species (dromedary camels, sheep, and goats) as well as cattle on some occasions. This environment may favor the heterologous infection of several strains of some viruses, particularly BVDV, among various species of animals.

This study aimed to explore the possibility of seroconversion of some species of animals such as dromedary camels, sheep, and goats living in various levels of contact with cattle to BVDV.

## Materials and Methods

### Ethical approval

The study was approved by the Ethics Committee of King Faisal University (approval no. KFU-REC/2020-12-36).

### Study period and location

The study was conducted from January 2018 to September 2020. This study was conducted in a semi-closed rearing system of a dromedary camel herd as well as in one of the large animal regional abattoirs in the eastern region of Saudi Arabia.

### Sampling of dromedary camels from two systems

We conducted this study in two systems (an abattoir and a semi-closed management system). We carried out abattoir surveillance among some dromedary camels admitted for slaughtering from 2014 to 2018 in the eastern region of Saudi Arabia. We also collected samples (sera, nasal, and rectal swabs) from one dromedary camel population containing various camel breeds (2019–2020). This came herd was housed in a wire fence sheltered house. This house was divided by wire meshes into several compartments to occupy several animals. Male animals were kept in individual pens and did not allow to mix with female animals except during the mating seasons (April–October) each year. Animals received food and water in a shared tray. The female animals from different breeds may allow mingling together in a large grooming area each day. Samples were collected from each animal from the jugular veins before starting the procedure of slaughtering.

### Collection of samples from sheep and goats

We collected samples (sera, nasal, and rectal swabs) from a herd of sheep and goats. Each species of animal is kept separately in designated wire fence partition pens. These animals were kept in wired fenced houses and separated from the dromedary camel herd with a wire mesh fence.

### Serum sample collection, preparation, and storage

The whole blood samples per each animal (camel, sheep, and goats) were collected without using any anticoagulant from the jugular vein as previously described [10–12]. The collected samples were kept at 4°C overnight. Separation of sera was carried out as previously described [11, 13, 14]. Simply, centrifugation of samples was done at 1398× *g*. We collected the clear supernatant sera and then transferred to another screw-capped 2 mL tube and then stored them at −20°C for further testing by enzyme-linked immunosorbent assay (ELISA) [14].

### Collection of the nasal and rectal swabs from camels, sheep, and goats

We collected both nasal and rectal swabs from dromedary camels in the semi-closed system as well as from sheep and goats. Nasal swab collections were carried out as previously described [13]. Simply, we introduced a clean, sterile cotton swab very deep into the nasal cavity to touch the back of the nasal septa. Each cotton swab was soaked in mucosal secretion. Swabs were transferred into a clean, sterile tube containing viral transport media as previously described [11, 13]. Each rectal swab was collected independently per animal by introducing the cotton swab into the rectum to collect some of the rectal secretions as well as fecal materials. The downstream procedure of processing both types of swabs is similar and was carried out as previously described by Hemida *et al*. [11] and Hemida *et al*. [13]. Swabs were transferred on ice to our laboratory for further processing. Each swab was shacked and vortexed vigorously, then centrifuged at 5590× *g* for 5 min at 4°C. We collected the supernatants in clean sterile 2 mL screw-capped tubes and stored them at −80°C for further testing.

### Preparation of serum and swab samples for ELISA

Each serum sample was heat inactivated by placing the tubes containing sera in a water bath for 30 min at 56°C to inactivate any known specific inhibitors. Then the sera were used to carry out the ELISA as described below;

### Sandwich ELISA for the detection of BVDV antigen in animal serum and swab samples

The BVDV protein 80 (P80) antigen ELISA kits (catalog no.: BVDC-10P) were used to detect BVDV antigen in serum and swab (nasal and rectal) samples collected from cattle, sheep, goats, and camels. A final dilution of 1:2 was prepared from each serum sample in dilution buffer. A volume of 100 μL of pre-diluted samples, undiluted nasal/rectal swabs eluate samples, and negative control or positive control serum was added to the wells of microtiter plates pre-coated with capture antibodies against the BVDV P80-125. After incubation for 60 min at 37°C, the plates were washed 5 times in washing buffer. A volume of 100 μL of the peroxidase-conjugated anti-BVDV P80-125 detection antibody was added to each well flowed by incubation for 30 min at 37°C. After three washes, the substrate-chromogen solution was added to the plates, and the plates were incubated for 30 min at 21°C in the dark. Finally, a stop solution was added to the plates, and the color density was measured using a spectrophotometer at 450 nm. The test was considered valid if the optical density (OD) value of the positive control was higher than 0.500 and the ratio of the mean values of the positive to the negative controls (ODpc/ODnc) was >3. For each tested sample, the percentage of sample/positive control (S/P %) was calculated. An S/P % of <35% was considered negative. An S/P % equal to or more than 35% was considered positive.

### Detection of BVDV-specific antibodies in animal serum using competitive (ELISA)

The commercial ID Screen**^®^** BVD p80 Antibody Competition ELISA kits (catalog no.: BVDC-10P, 310 rue Louis Pasteur 34790 Grabels, France) were also used to test serum samples for the presence of BVDV antibodies. A microtiter plate containing all control and tested samples were prepared before transferring them into the ELISA plate using a multichannel pipette. A final dilution of 1:100 was prepared from each serum sample in dilution buffer. A volume of 100 μL of pre-diluted samples, negative control, or positive control serum was added to the wells of microtiter plates pre-coated with the BVD viral protein P80-125. After incubation for 45 min at 37°C, the plates were washed 3 times with 300 μL of the washing buffer. A 100 μL volume of peroxidase-conjugated anti-BVDV P80-125 antibody was added to each well, followed by incubation for 30 min at 21°C. After three washes, the substrate-chromogen solution was added to the plates, and the plates were incubated for 30 min at 21°C in the dark. Finally, a stop solution was added to the plates, and the color density was measured using a spectrophotometer at 450 nm. The test was considered valid if the OD value of the negative control (ODnc) was >0.7 and the mean value of the positive control ODpc was <30% of the ODnc values higher than 3. For each sample, the competition percentage (S/N %) was calculated by dividing the OD value of the sample by the OD value of the negative control and multiplication of the result by 100 (OD-sample/ODnc 100×). A competition percentage equal to or <40% was considered positive. A competition percentage equal to or more than 50% was considered negative. A competition percentage between 40% and 50% was considered doubtful.

### Statistical analysis

The seroprevalence of BVDV in different species of animals (dromedary camels, goats, and sheep) was calculated using the exact binomial confidence intervals (CIs) of 95% by the use of the Binom test of R programming analytical procedure (https://www.r-project.org/foundation/Rfoundation-statutes.pdf). The associations of BVD in camels with different risk factors were evaluated using the Cochran-Armitage trend test [15]. The strength of associations was assessed through phi and Cramer’s V value using the R programming analytical procedure. The fit of the multivariable logistic regression model was evaluated by the application of the Hosmer–Lemeshow goodness-of-fit test [16]. Both the univariate and multivariate logistic regression tests were applied.

## Results

### Detection of BVDV in sera of dromedary camels, sheep, and goats

**Figure-1:**
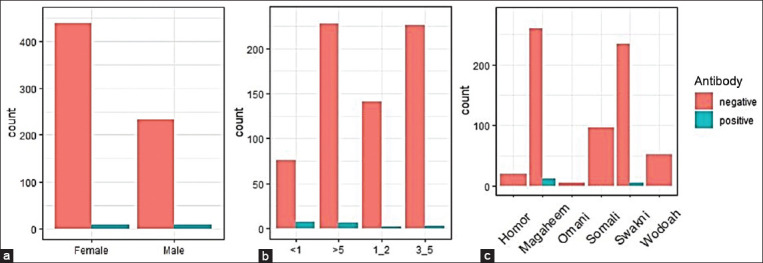
The bovine viral diarrhea virus (BVDV) competitive enzyme-linked immunosorbent assay results on serum samples collected from the abattoir surveillance of dromedary camels 2014–2018. Analysis of some risk-associated factors for BVDV seroconversion in abattoir surveillance among some dromedary camels 2014–2018 (a) Gender, (b) age, and (c) breed.

Our abattoir surveillance showed that 38/906 (4.1%) of the tested animals were positive for the BVDV antibodies (Table-1). Meanwhile, 8/106 (7.5%) serum samples collected from the semi-closed dromedary camel herd were positive (Table-1). None of the tested 21 sheep samples was positive; however, 3/84 samples from goats (3.5%) were positive. Our overall seroprevalence in camels, sheep, and goats showed that 49/1117 serum samples (4.38%) were positive for BVDV antibodies (Table-1 and Figure-1).

**Table-1:** Results of the seroprevalence of bovine viral diarrhea virus in dromedary camels, sheep, and goats.

S. No.	Animal species	Total	(+Ve)	(-Ve)	% prevalence
1	Dromedary camels				
	Abattoir surveillance	906	38	868	4.1
	Semi-closed dromedary camel population	106	8	98	7.5
2	Sheep	21	0	21	0
3	Goats	84	3	81	3.5
#	Total	1117	49	1068	4.38

### Detection of BVDV antigen in the nasal swabs of some dromedary camels, sheep, and goats

We tested 106, 21, and 84 animals (nasal and rectal swabs) from dromedary camels in the semi-closed herd, sheep, and goats. All the tested swabs were negative (Table-2).

**Table-2:** BVD antigen detection in serum and nasal swab samples from camel, sheep, and goats.

Animal species	Total animals	(+Ve)	(-Ve)	% prevalence
Camels	106	0	106	0
Sheep	21	0	21	0
Goats	84	0	84	0

BVD=Bovine viral diarrhea

### Exploring some associated risk factors for the BVDV in dromedary camels

We analyzed some factors (age, sex, and breed) associated with BVDV infection in the abattoir surveillance of some dromedary camels in the eastern region of Saudi Arabia from 2014 to 2018. We used the data from 906 animals to explore the association of some factors with the prevalence of BVDV seroconversion in dromedary camels. Both Table-3 and Figure-1 show a descriptive analysis of variables used to predict the seroprevalence of BVDV in dromedary camels. Our results show a weak association between the seroprevalence of BVDV and the age of animals (phi coefficient and Cramer’s V = 0.139). A weak association with breed V = 0.109. However, phi coefficients/Cramer’s V of 0.05 indicated no association between the seroprevalence of BVDV in camels and the gender of the animal. The seroprevalence of BVDV in camels was determined in 689 serum samples that were obtained from six different breeds of camels (Magaheem, Homor, Omani, Somali, Swakni, and Wodoah), and was of ages that ranged from <1 to 12 years (Table-4). Of 689 tested camel serum samples, 18 sera (2.6%) showed antibodies by indirect ELISA (95% CI 0.015–0.04, p < 0.001). There were statistically significant differences among the age groups (p = 0.013) using Fisher’s test. The results of the univariate logistic regression test (Table-5) showed that animals of age <5 have a lower significant infection risk of BVD by 71% (OR: 0.29, CI: 0.09–0.89, p = 0.028) compared to camels with age >1. However, 1–2-year-old animals have a significant decrease in the risk of infection of BVD by 85% (OR: 0.15, CI: 0.02–0.66, p = 0.022) compared to camels younger than 1-year-old. Furthermore, in the case of animals aged 3–5, there is a significant decrease in the risk of infection of BVDV by 86% (OR: 0.14, CI: 0.03–0.53, p = 0.006) compared to camels with age >1. The results of the multivariate logistic regression test (Table-6) showed that animals of 1–2 years old have a significant decrease in the risk of infection of BVDV by 84% (OR: 0.16, CI: 0.02–0.73, p = 0.030) compared to younger camels <1-year-old. In addition, in the case of animals aged 3–5 years old, there was a significant decrease in the risk of infection of BVDV by 82% (OR: 0.18, CI: 0.04–0.72, p = 0.020) compared to camels >1-year-old.

**Table-3:** Descriptive analysis of variables used to predict the seroprevalence of BVDV in dromedary camels.

Variable	Category	No. of camels	Distribution (%)
Age (year)	<1	83	12
	>5	234	34
	1–2	143	21
	3–5	229	33
Sex	Female	447	65
	Male	242	35
Breed	Homor	21	3
	Magaheem	272	39
	Omani	6	1
	Somali	97	14
	Swakni	240	35
	Wodoah	53	8

BVDV=Bovine viral diarrhea virus

**Table-4:** Descriptive analysis of variables used to predict the seroprevalence of BVD in camels showing Fisher p-value.

Variable	Category	No. of camels	No. of positive	Prevalence (%)	p-value
Age	<1	83	7	8.4	0.013
	>5	234	6	2.6	
	1–2	143	2	1.4	
	3–5	229	3	1.3	
Sex	Female	447	9	2	0.213
	Male	242	9	3.7	
Breed	Homor	21	0	-	0.188
	Magaheem	272	12	4.4	
	Omani	6	0	-	
	Somali	97	0	-	
	Swakni	240	6	2.5	
	Wodoah	53	0	-	

p ≤ 0.05 is statistically significant, BVD=Bovine viral diarrhea

**Table-5:** Univariable logistic regression analysis of the association of BVD in camels with different risk factors.

Variable	Category	Negative	Positive	OR (univariable)	CI	p-value
Age	<1	76 (91.6)	7 (8.4)	-		
	>5	228 (97.4)	6 (2.6)	0.29	0.09–0.89	0.028
	1–2	141 (98.6)	2 (1.4)	0.15	0.02–0.66	0.022
	3–5	226 (98.7)	3 (1.3)	0.14	0.03–0.53	0.006
Sex	Female	438 (98.0)	9 (2.0)	-		
	Male	233 (96.3)	9 (3.7)	1.88	0.72–4.88	0.187
Breed	Homor	21 (100.0)	0 (0.0)	-		
	Magaheem	260 (95.6)	12 (4.4)	39440100.10	0.00-NA	0.996
	Omani	6 (100.0)	0 (0.0)	1.00	0.00-∞	1.000
	Somali	97 (100.0)	0 (0.0)	1.00	0.00-∞	1.000
	Swakni	234 (97.5)	6 (2.5)	21911166.72	0.00-NA	0.997
	Wodoah	53 (100.0)	0 (0.0)	1.00	0.00-∞	1.000

p ≤ 0.05 is statistically significant, BVD=Bovine viral diarrhea, OR=Odds ratio, CI=Confidence interval.

**Table-6:** -Multiple logistic regression analysis of potential risk factors associated with BVD seropositivity in camels.

Variable	Category	β	SE	OR (multivariable)	CI	p-value
Intercept		−19.58	3724			>0.99
Age	<1	Reference	Reference	-		
	>5	−1.14	0.627	0.32	0.09–1.10	0.021
	1–2	−1.80	0.833	0.16	0.02–0.73	0.023
	3–5	−1.705	0.735	0.18	0.04–0.72	0.011
Sex	Female	Reference	Reference			
	Male	0.42942	0.52955	1.54	0.53–4.37	0.417
Breed	Homor	Reference	Reference	-		
	Magaheem	17.36	3724	34693943	0.00-NA	0.996
	Omani	0.130	8093	1.14	0.00-∞	1.000
	Somali	0.042	4115	1.04	0.00-∞	1.000
	Swakni	17.01	3724	24621607.16	0.00-NA	0.996
	Wodoah	−0.019	4410	0.98	0.00-∞	1.000

p ≤ 0.05 is statistically significant, BVD=Bovine viral diarrhea, SE=Standard error, OR=Odds ratio, CI=Confidence interval.

## Discussion

BVDV is still one of the major viral concerns for many animal species – mainly cattle, sheep, and goats. The virus was recently reported in many other wildlife animals, such as white-tailed deer, free-range deer, and captive angulates in a zoo [8, 17, 18]. Several BVDV infections were reported in dromedary camels from several African countries such as Sudan, Egypt, and Algeria [19–21]. This is in addition to other outbreaks reported in Asian countries such as the Kingdom of Saudi Arabia, India, and Iran [12, 22, 23].

Meanwhile, sheep and goats may also play important roles in the sustainability of BVDV in the environment, thus posing a risk of transmitting the virus to other species of animals, particularly cattle. In many regions across the world, cattle, sheep, and goats used to graze and share the pasture for a long time. This may favor the transmission of not only BVDV among these species but also many other viral, bacterial, and parasitic diseases. BVDV has been reported in sheep and goats in many regions of the world [24–26].

This is in addition to several outbreaks of BVDV reported in sheep and goats in many regions across the globe [5, 12, 24, 27]. Several approaches have been adopted for the diagnosis of BVDV infection in various animal species [28–33]. This may include viral isolation using various cell lines, identification of viral antigens in various body fluids and excretions, detection, and quantification of viral antibodies, particularly using ELISA techniques as well as detection of the viral nucleic acids [8, 34]. Several types of ELISA techniques were used to detect the viral antigens or antibodies (competitive, inhibition, and sandwich) [35]. The performance of these types of ELISAs was good compared to other laboratory techniques such as virus neutralization assay and the agar gel immunodiffusion test [36].

Our results showed the seroconversion of dromedary camels both in the abattoir surveillance and semi-closed animal population and goats (Figure-1 and Tables-1–3). As long as there are no active vaccination programs for BVDV in the KSA in dromedary camels, this strongly suggests that those animals are exposed to an active field infection. Although none of the tested samples from sheep showed positive antibodies against BVDV, this may be related to the small sample size of this species of animals. This is in contrast to the detection of BVDV antibodies in sera of several populations of sheep from different countries in Africa and Asia [12, 24].

Our results show that both genders (males and females) are equally susceptible to BVDV infection. There is no statistically significant difference between males and females in the context of BVDV infection or seroconversion (Tables-3–6). Our results show that the age of the animals could be one of the risk-associated factors in the context of BVDV infection (Tables-3–6). Young animals seem to be more susceptible to viral infection than adult animals [37]. However, the older animals have a much higher magnitude of viral antibodies than the naïve animals. One explanation for this pattern is the possibility of multiple rounds of infections per animal throughout its life. This could increase the level of antibodies in their sera. ELISA technique was used to distinguish the herd of animals that have persistently infected animals from free herds based on the antibody [5, 18, 38, 39]. Younger calves with high antibody titers measured by ELISA could be an indicator for the PI animal population [25, 40].

There are some associated risk factors that enhance BVDV infection in various species of animals, including cattle, sheep, goats, and camels. These factors include the introduction of new animals to some herds, and sharing the grazing land in pasture [41]. Other studies showed a high seroprevalence of BVDV in cattle than in water buffalo, suggesting that the species of animals may play some roles in the susceptibility/resistance to the virus [42]. Meanwhile, the latter study showed no significant correlation between gender, age, and breed with BVDV infection [42]. This study is aligned with our findings that gender may not represent a risk factor for BVDV infection in contrast to age. Our study showed that younger animals could be much more susceptible to the virus infection. Further large-scale studies include several breeds and a wide age range of animals to consolidate the age as a risk factor for BVDV infection.

Further large-scale molecular and serological surveillance for the BVDV among various species of animals, particularly dromedary camels, sheep, and goats that live in close contact with cattle, is highly recommended. This will provide up-to-date information about the dynamics and epidemiology of BVDV among various species of animals.

This study explored the possibility of BVDV seroconversion in some animals, particularly dromedary camels, sheep, and goats, and some associated risk factors. We conclude that the investigated species of animals may play some important roles in the transmission cycle of BVDV and its suitability in certain animal populations. Those animals may pose a great risk to the cattle industry when raised in their close proximity. We also conducted some age stratifications of the dromedary camels to assess the impacts of age on the seroprevalence of BVDV. Our findings clearly confirmed that the naïve animals <1-year-old are much more at-risk group for infection compared to the adult animals, which may have a high magnitude of antibody concentration in their sera. This highlights the mandate of the implementation of active vaccine programs against BVDV in these animal species to reduce the risk of infection among these animals as well as to reduce the spillover of the virus from these species to cattle.

## Conclusion

Our study shows the seroprevalence in some dromedary camels as well as goats for the BVDV. Since these animals were not vaccinated against BVDV, they should be exposed to the viral infection at a certain time of their life. Thus, we conclude that at least dromedary camels and goats play roles in transmitting the BVDV.

## Data Availability

The supplementary data can be available from the corresponding author on a reasonable request.

## Authors’ Contributions

MGH and AIA: Conceived and designed the study. MGH, AIA, JH, MK, AAGA, BF, and AS: Performed experiments, data analysis, and drafted and revised the manuscript. All authors have read and approved the final manuscript.
